# Allantoin improves methionine-choline deficient diet-induced nonalcoholic steatohepatitis in mice through involvement in endoplasmic reticulum stress and hepatocytes apoptosis-related genes expressions 

**DOI:** 10.22038/ijbms.2019.33553.8012

**Published:** 2019-07

**Authors:** Tahereh Komeili Movahhed, Azam Moslehi, Mohammad Golchoob, Shima Ababzadeh

**Affiliations:** 1 Cellular & Molecular Research Center, Qom University of Medical Sciences, Qom, Iran; 2 Student Research Committee, Qom University of Medical Sciences, Qom, Iran

**Keywords:** Allantoin, Liver, Non-alcoholic steatohepatitis, PPARα, SREBP1c, Steatosis

## Abstract

**Objective(s):**

Non-alcoholic steatohepatitis (NASH) is defined by steatosis and inflammation in the hepatocytes, which can progress to cirrhosis and possibly hepatocellular carcinoma. However, current treatments are not entirely effective. Allantoin is one of the principal compounds in many plants and an imidazoline I receptor agonist as well. Allantoin has positive effects on glucose metabolism and inflammation. In this study, the effects of allantoin on the NASH induced animals and the pathways involved have been evaluated.

**Materials and Methods:**

C57/BL6 male mice received saline and allantoin as the control groups. In the next group, NASH was induced by the methionine-choline-deficient diet (MCD) for eight weeks. In the NASH+allantoin group, allantoin was injected four weeks in the mice feeding on an MCD diet. Histopathological evaluations, serum analysis, ELISA assay, and real-time RT-PCR were performed.

**Results:**

Allantoin administration decreased serum alanine aminotransferase (ALT), cholesterol, low-density lipoprotein (LDL), hepatic lipid accumulation, and liver tumor necrosis factor (TNFα) level. Also, treatment with allantoin down-regulated the gene expression of glucose-regulated protein 78 (GRP78), activating transcription factor 6 (AFT6), TNFα, sterol regulatory element binding proteins 1c (SREBP1c), fatty acid synthase (FAS), Bax/Bcl2 ratio, caspase3, and P53. On the other hand, peroxisome proliferator-activated receptor alpha (PPARα), apolipoprotein B (Apo B), and acetyl-coenzyme acetyltransferase 1 (ACAT1) gene expression increased after allantoin injection.

**Conclusion:**

This study indicated that allantoin could improve animal induced NASH by changes in the expression of endoplasmic reticulum stress-related genes and apoptotic pathways.

## Introduction

Non-alcoholic fatty liver disease (NAFLD) is one of the most common liver diseases worldwide and is identified by the excess accumulation of triglycerides in the hepatocytes (steatosis). Subsequently, it can progress to non-alcoholic steatohepatitis (NASH), a severer feature of the NAFLD designated as steatosis, hepatocyte inflammation with or without fibrosis, then cirrhosis, and probably hepatocellular carcinoma (1). It has been estimated that NAFLD involves approximately one-third of the population in Western countries, 20% of whom develop into NASH (2). In another study, the global prevalence of NASH has been estimated at 24.4% (3). However, the basic mechanisms underlying the progression of NASH are not mostly clear. Obesity and insulin resistance are known as the primary etiology of NAFLD/NASH (4). Increased hepatic saturated and unsaturated fatty acid levels provide lipid accumulation and steatosis. Activation of hepatic resident Kupffer cells, neutrophils, and other innate immune cells then leads to the release of cytokines/chemokines and production of inflammation (5). Moreover, ER stress, gut toxins, adipokines imbalance, increased oxidative stress, and some drugs are considered as other NASH inducing factors (6, 7). Despite high prevalence and liver injuries, therapeutic options for NASH treatment are limited. Nowadays, lifestyle change, weight loss, increased physical exercise, and lipid-lowering drugs are recommended to patients with NASH (8). Therefore, widespread research on the numerous pharmacologic and natural compounds is being carried out.

Allantoin is one of the main compounds in many plants such as *yam, Nymphaea nucifera *rhizome*, *sugar beet, and* leguminous,* and is a natural, safe, and non-toxic compound (9, 10). The wound healing and tissue regeneration effects of allantoin are already well known (11, 12). It has also been reported that allantoin decreases interleukine-4 (IL-4), IL-5, and immunoglobulin E (Ig-E) levels and leukocyte cells in ovalbumin (OVA)-induced lung inflammation (13). A study showed that allantoin had nociceptive and anti-inflammatory effects on formalin-induced nociception test (14). Allantoin also improved cognitive function and neurogenesis in mice hippocampus (15). Moreover, allantoin activates imidazoline I receptor (IR) in animal and cell lines (16). Recent studies have shown allantoin affects metabolic function. For example, Chung *et al*. have stated that allantoin alleviates obesity and hyperlipidemia via IR1 activation in mice (17). 

To complement the previous studies, this study was designed to assess the effects of allantoin on MCD diet-induced NASH in mice. In this experiment, the MCD diet was used to induce NASH. The most widely used diet for NASH induction is the methionine-choline-deficient diet (MCD) in mice. This diet is enriched with high sucrose (>40%) and moderate fat (10–20%), which provide insulin resistance. This method causes weight loss and severe steatosis in the liver after eight weeks due to the reduced lipid export from the liver and lipid accumulation (18). Since ER stress promotes NASH via changes in lipid metabolism-related gene expression (7), ER stress inducer genes (GRP78 and ATF6) and expression of lipid metabolism-related genes (SREBP1c, PPARα, FAS, Apo B, and ACAT1) and effects of allantoin on them were evaluated. One of the untreated aspects of NASH is liver apoptosis. Hence, allantoin effects on the apoptosis pathway genes were evaluated (P53, Bax, bcl2, and caspase3). 

## Materials and Methods

***Experimental procedures***

C57/BL6 male mice weighing 25–27 g (Pasteur Institute, Tehran, Iran) were used in this study. Animals were housed in a temperature-controlled room under a 12:12 hr light/dark cycle with free access to food and tap water. The animal care and experimental procedure were approved in accordance with the Guidelines for Animal Care and Use at the Qom University of Medical Sciences (IR.MUQ.REC.1394.156). All mice were randomly divided into four equal groups (n=6). The control group had access to a standard diet for eight weeks and received normal saline (IP) for four weeks. The allantoin group had access to a standard diet for eight weeks and received allantoin (5 mg/kg, IP) for four weeks daily (16). The NASH group received the MCD diet for eight weeks to induce NASH (19), and NASH+ allantoin group received the MCD diet for eight weeks to induce NASH and received allantoin (5 mg/kg, IP) for four weeks, daily. After this period, the animals were anesthetized with sodium pentobarbital (20). The abdomen was excised via a midline incision. The liver was immediately removed, weighed, and washed in ice-cold physiological saline. Blood samples were directly collected from the heart. A part of the liver was dissected and kept at –70 ^°^C.

***Histological study***

A part of the median lobe was dissected and fixed in 10% buffered formaldehyde solution. 5 μm thick sections from paraffin-embedded liver tissue were then obtained for hematoxylin and eosin (H&E) staining. Fresh frozen tissue sections from another part of the median lobe were obtained for Oil red O staining. An expert pathologist, blind to the experiment determined hepatic steatosis, hepatocytes ballooning, and lobular inflammation based on scoring system of Kleiner *et al*. (21). Oil red O staining was performed to show the content of lipid droplets.

***Serum lipids and enzymes measurement***

Serum was obtained from centrifuged blood samples (3500 rpm for 20 min), and total cholesterol, triglyceride, LDL, and alanine aminotransferase (ALT) levels were measured.

***Real-time RT-PCR***

Total RNA was extracted from frozen tissue samples using the RNX-plus Kit (Cinnagen, Tehran, Iran) according to the manufacturer’s instructions. Briefly, 1 ml of ice-cold RNXTM –PLUS solution was added to homogenized samples. After 5 min incubation at room temperature, 200 μl of chloroform was added. The mixture was then incubated on ice and centrifuged at 12000 rpm at 4 ^°^C for 15 min. The aqueous phase was dissected, and an equal volume of isopropanol was added, followed by incubation and centrifugation. Afterward, the supernatant was discarded, and 75% ethanol was added, and the mixture was centrifuged at 4 ^°^C for 8 min at 7500 rpm. Finally, the pellets were allowed to dry at room temperature and dissolved in 50 μl of DEPC treated water. The quantity and purity of the RNA samples were measured by a Nanodrop spectrophotometer (Thermo Scientific, USA). Complementary DNAs (cDNA) were made from mRNA templates for qRT-PCR using the Pocket Script RT premix (Sinaclon, Iran). Briefly, at first, RNA-primer mixture was prepared as follows: 5 μg of total RNA and 1 μl of oligo d(T) primer solution were dissolved in DEPC treated water and diluted to 10 μl. The mixture was incubated at 65 °C for 5 min and chilled on ice. cDNA synthesis mixture was then prepared as follows: 0.5 μl of MMLV reverse transcriptase, 2 μl of 10X buffer MMLV, 0.5 μl of RNase inhibitor, and dNTP were mixed in DEPC treated water. cDNA mixture was added to RNA-primer mixture. Afterward, the mixture obtained was centrifuged and incubated at 42 °C for 60 min. The reaction was terminated by incubation at 85 °C, and the product was cooled and stored. The synthesized cDNAs were used as templates for real-time PCR. For real-time quantification, a 20 μl reaction mixture consisting of 10 μl of 2× SYBR Green master mix (Biofact), 1 μl of template, 1 μl of forward and 1 μl of reverse specific primers, and 8 μl of DEPC of treated water was prepared. The initial denaturing temperature was 95 ^°^C for 15 min, followed by 40 cycles at 95 ^°^C for 20 sec, 59 ^°^C for 20 sec, and 72 ^°^C for 30 sec. Glyceraldehyde-3-phosphate dehydrogenase (GAPDH) was used as an internal control. Melting curve analysis was conducted at temperatures between 60 ^°^C and 95 ^°^C. The quantitation of data was performed using the comparative 2^-ΔΔCt^ method. Primers for the genes studied were designed by primer3 software (v. 0.4.0) (http://primer3.ut.ee) and were based on sequences found in the ensemble. Primer sequence homology and total gene specificity were confirmed with BLAST analysis (www.ncbi.nlm.nih.gov/blast). The primers sequences were as follows:

***Tissue TNF-α determination***

Weighed samples of the liver tissues (approximately 0.5 g) were fully homogenized. Then supernatants were separated for TNF-α assay using an immunometric enzyme immunoassay (EIA) kit (ADI-900-047-Enzo Life Sciences).

***Statistical analysis***

Data normality was checked by the Kolmogorov–Smirnov test. Data were expressed as mean±SEM. Statistical analysis was performed using the chi-square test for histopathological results and one-way analysis of variances (ANOVA) and Tukey’s *post hoc* test for other findings using SPSS. *P*<0.05 was considered statistically significant.

## Results

***Effects of allantoin on histopathological evaluations***

**Figure 1:**
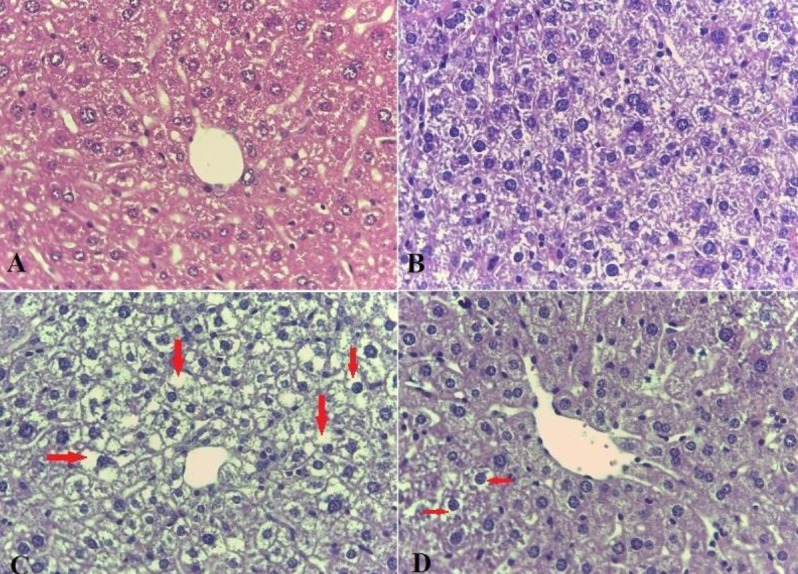
Histological findings of liver tissues after hematoxylin and eosin (H&E) staining (magnification; ×200) in different experimental groups. A; Control group: Normal liver histology, B; Allantoin group: Normal liver histology, C; NASH group: showing steatosis and ballooning degradation (Empty spaces in the cell indicate fat accumulation and enlargement of cells), D; NASH + allantoin group: showing lower steatosis and lobular inflammation (lower empty spaces)

**Figure 2.**
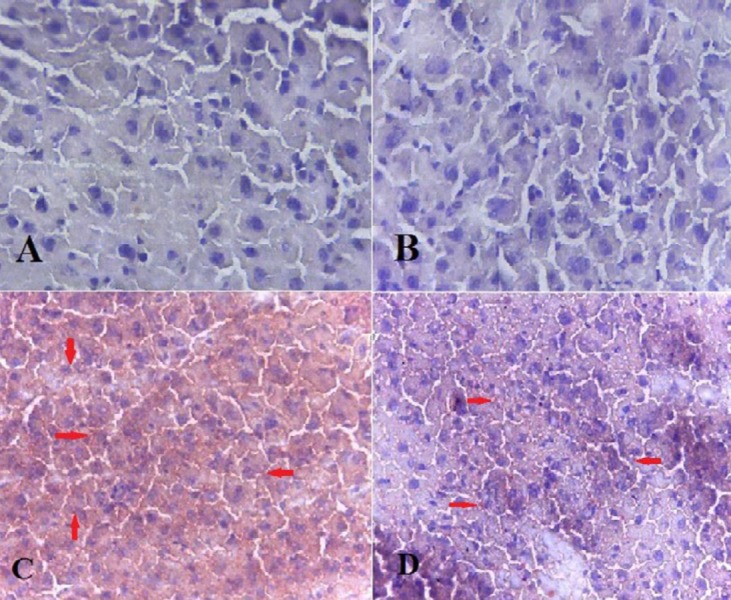
Histological findings of liver tissues after Oil red O staining (magnification; ×200) in different experimental groups. A; Control group: Normal liver histology, B; Allantoin group: Normal liver histology, C; NASH group: showing steatosis (red spots), D; NASH + allantoin group: showing lower steatosis (red spots)

In this experiment, both H&E and Oil red O staining were performed. Our findings revealed excessive lipid droplet accumulation (red points in Oil red O staining and empty spaces in H&E staining), cellular ballooning, and lobar inflammation in MCD induced mice. However, steatosis, edema, and inflammation were significantly lowered in the NASH+allantoin group (Figures 1, 2 and Table 1). 

***Effects of allantoin on liver index measurement***

As expected, the results showed that liver index (body weight/liver weight×100) significantly increased in the NASH group compared with the control group while allantoin administration markedly decreased liver index compared with the NASH group (Table 2).

***Effects of allantoin on biochemical analysis***

As noted in Table 2, blood sample analysis showed that the ALT level significantly increased in the NASH group compared with controls. However, administration of allantoin caused a marked decrease in the ALT serum level in the NASH+allantoin group compared with the NASH group. Serum level of TG also significantly decreased in the NASH group compared with the control, as expected from the MCD diet method. However, TG serum level significantly increased in the NASH+allantoin group compared with the NASH group. The results showed that the serum level of cholesterol markedly increased in the NASH group compared with the control group. Allantoin treatment after four weeks significantly decreased cholesterol levels in the NASH+allantoin group compared with the NASH group. In the NASH group, LDL serum level also significantly increased compared with the control group. LDL serum level decreased remarkably in the NASH+allantoin group compared with the NASH group.

***Effects of allantoin on gene expression of glucose-regulated protein 78 (GRP78) and activating transcription factor 6 (ATF6) in the NASH induced mice***

**Figure 3:**
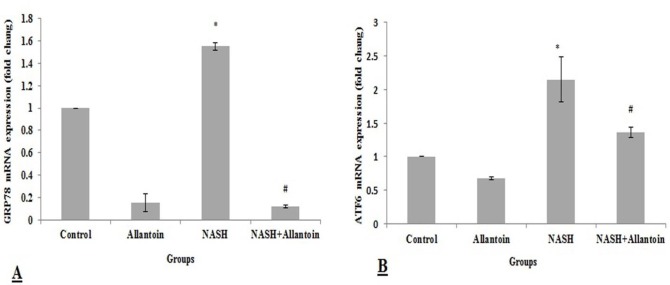
mRNA expression of glucose-regulated protein 78 (GRP78) and activating transcription factor 6 (ATF6) in different experimental groups. (Mean±SEM, N=6), * *P*<0.01 compared with the control group, # *P*<0.001 compared with the NASH group for GRP78 and # *P*<0.05 compared with the NASH group for ATF6 (one-way ANOVA followed by Tukey’s *post hoc* test). NASH: Non-alcoholic steatohepatitis

This study showed that GRP78 gene expression, as ER stress chaperon, significantly increased in the NASH group compared with the control group (1.55±0.032 vs 1, *P*<0.01) while it significantly decreased in the NASH+allantoin group compared with the NASH group (0.123±0.013 vs 1.55±0.032, *P*<0.001) (Figure 3A). Similarly, AFT6 gene expression, one of the downstream signaling cascades, significantly increased in the NASH group compared with the control group (2.15±0.33 vs 1, *P*<0.01), but decreased in the NASH+allantoin group compared with the NASH group (1.36±0.078 vs 2.15±0.33, *P*<0.05) (Figure 3B).

***Effects of allantoin on gene expression of SREBP1c and PPARα in the NASH induced mice***

**Figure 4:**
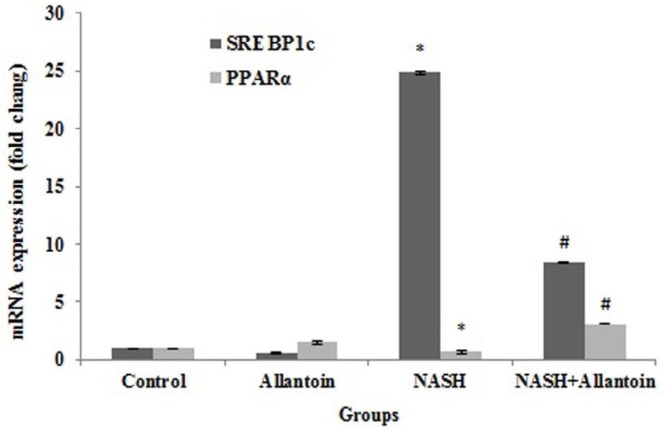
mRNA expression of sterol regulatory element binding proteins 1c (SREBP1c) and peroxisome proliferator-activated receptor alpha (PPARα) in different experimental groups. (Mean±SEM, N=6), * *P*<0.001 compared with the control group, # *P*<0.01 compared with the NASH group (one-way ANOVA followed by Tukey’s *post hoc* test)

SREBP1c and PPARα are two transcriptional factors in the lipid metabolism pathway, which change in ER stress and NASH induction. Gene expression of SREBP1c significantly increased in the NASH group compared with the control group (24.85±0.05vs 1, *P*<0.001), as expected. After four weeks of allantoin administration, SREBP1c significantly decreased in the NASH+allantoin group compared with the NASH group (8.42± 0.06vs 24.85±0.06, *P*<0.001). On the contrary, MCD diet markedly decreased PPARα gene expression compared with the control group (0.72±0.13 vs 1, *P*<0.01) while allantoin treatment significantly increased PPARα in the NASH+allantoin group compared with the NASH group (3.09±0.01vs 0.72±0.13, *P*<0.01) (Figure 4).

***Effects of allantoin on gene expressions of the enzymes involved in the NASH induced mice***

**Figure 5:**
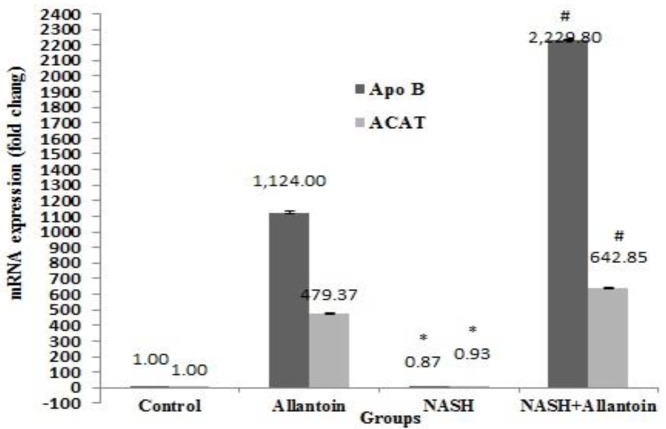
mRNA expression of apolipoprotein B (ApoB) and acetyl-coenzyme acetyltransferase (ACAT1) in different experimental groups. (Mean±SEM, N=6), * *P*<0.05 compared with the control group, # *P*<0.001 compared with the NASH group (one-way ANOVA followed by Tukey’s *post hoc* test)

**Figure 6:**
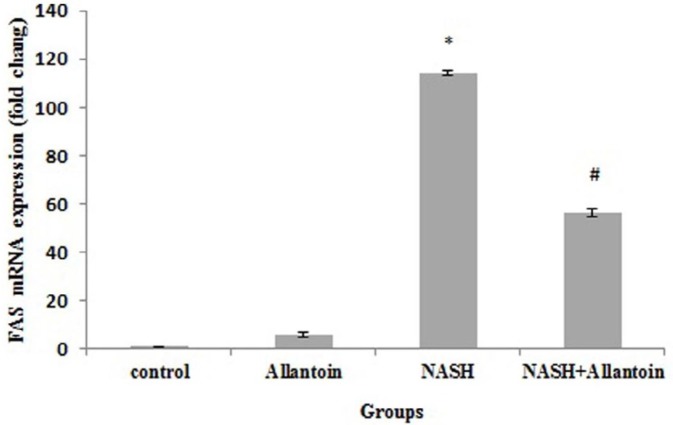
mRNA expression of fatty acid synthase (FAS) in different experimental groups. (Mean±SEM, N=6), * *P*<0.001 compared with the control group, # *P*<0.01 compared with the NASH group (one-way ANOVA followed by Tukey’s* post hoc* test)

The effects of allantoin on apolipoprotein B (Apo B), acetyl-coenzyme acetyltransferase (ACAT1), and fatty acid synthase (FAS) were studied to gain more accurate results. The MCD diet in mice significantly decreased Apo B gene expression compared with the control group (0.87±0.03 vs 1, *P*<0.05) while in the NASH+allantoin group it significantly increased compared with the NASH group (2,229.8±5.87 vs 0.87±0.03, *P*<0.001). The gene expression of ACAT1 enzyme significantly decreased in the NASH group compared with the control group (0.93±0.05 vs 1, *P*<0.05). However, allantoin administration in MCD mice significantly increased ACAT1 compared with the NASH group (642.85±4.70vs 0.93±0.05, *P*<0.001) (Figure 5). The results showed a marked increment of FAS gene expression in the NASH group compared with the control group (114.01±1.06 vs 1, *P*<0.001). Nevertheless, allantoin significantly lowered FAS gene expression compared with the NASH group (56.48±1.78 vs 114.01±1.06, *P*<0.01) (Figure 6)

***Effects of allantoin on the inflammatory cytokine expressions in the NASH induced mice***

**Figure 7:**
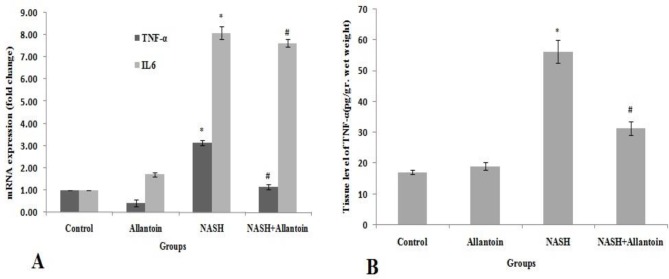
A: mRNA expression of TNFα and IL6 in different experimental groups. (Mean±SEM, N=6), * *P*<0.01 compared with the control group, # *P*<0.01 compared with the NASH group (one-way ANOVA followed by Tukey’s post hoc test); B: TNFα level of the liver tissue in different experimental groups. (Mean±SEM, N=6, pg/gr wet weight), * *P*<0.01 compared with the control group, # *P*<0.01 compared with the NASH group (one-way ANOVA followed by Tukey’s *post hoc* test)

As presented in Figure 7A, real-time PCR analysis showed that TNFα mRNA significantly increased in the NASH group compared with the control group (3.14±0.1 vs 1, *P*<0.01) while in the NASH+allantoin group it significantly decreased compared with the NASH group (1.57±0.11 vs 3.14±0.1, *P*<0.01). Our findings indicated that another inflammatory cytokine, i.e., IL6 gene expression, significantly increased in the NASH group compared with the control group (8.09±0.28 vs 1, *P*<0.01). Interestingly, allantoin treatment did not change compared with the NASH group.

***Effects of allantoin on the TNF-α measurement of the liver in the NASH induced mice***

This study revealed that TNF-α significantly increased in the NASH group compared with the control group (56.3±3.6 vs 17.2±0.52, pg/gr wet weight, *P*<0.01). However, allantoin administration significantly lowered TNF-α in the liver tissue compared with the NASH group (31.4±2.2 vs 56.3±3.6, pg/gr wet weight, *P*<0.01) (Figure 7B).

***Effects of allantoin on P53 mRNA expression in the NASH induced mice***

**Figure 8:**
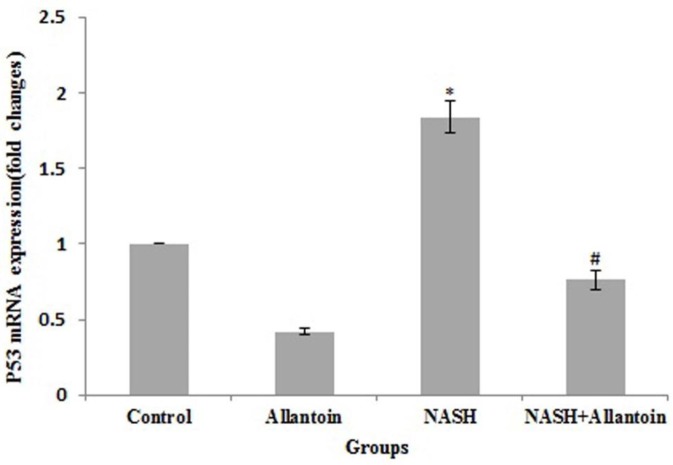
mRNA expression of P53 in different experimental groups. (Mean±SEM, N=6), * *P*<0.01 compared with the control group, # *P*<0.01 compared with the NASH group (one-way ANOVA followed by Tukey’s *post hoc* test)

Apoptosis is one of the important aspects of NASH that has not yet been treated. The results obtained showed a marked increase of P53 gene expression in the NASH group compared with the control group (1.84±0.11 vs 1, *P*<0.01). Nevertheless, allantoin treatment significantly decreased P53 gene expression compared with the NASH group (0.76±0.06 vs 1.84±0.11, *P*<0.01) (Figure 8).

***Effects of allantoin on the Bax/Bcl-2 ratio and mRNA expressions in the NASH induced mice***

**Figure 9:**
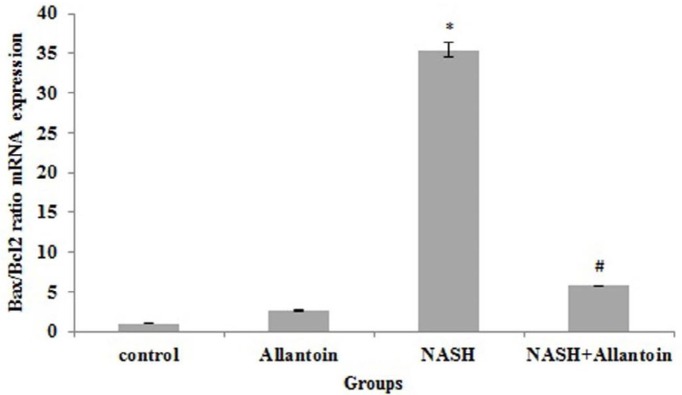
mRNA expression of the Bax/Bcl2 ratio in different experimental groups. (Mean±SEM, N=6), * P<0.001 compared with the control group, # *P*<0.001 compared with the NASH group (one-way ANOVA followed by Tukey’s *post hoc* test)

Our study revealed that in the NASH induced mice, Bax/Bcl2 ratio increased significantly compared with the control group (35.42±0.97 vs 1, *P*<0.001) while in allantoin treated mice it significantly dropped compared with the NASH group (7.55±0.005 vs 35.42±0.97, *P*<0.001) (Figure 9).

***Effects of allantoin on caspase3 mRNA expression in the NASH induced mice***

**Figure 10:**
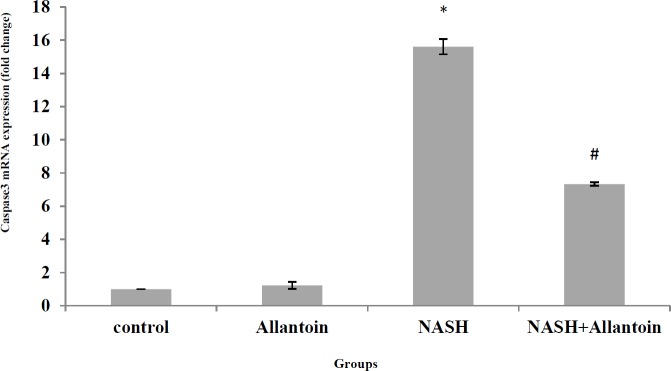
mRNA expression of caspase3 in different experimental groups. (Mean±SEM, N=6), * *P*<0.001 compared with the control group, # P<0.01 compared with the NASH group (one-way ANOVA followed by Tukey’s *post hoc *test

As noted in Figure 10, MCD diet in mice significantly increased caspase3 mRNA expression in the NASH group compared with the control group (15.61±0.45 vs 1, *P*<0.001). However, treatment with allantoin significantly lowered caspase3 mRNA expression compared with the NASH group (7.33±0.11 vs 15.61±0.45, *P*<0.01).

## Discussion

This study showed that allantoin attenuated ER stress-related genes; lipid accumulation and inflammation in the hepatocytes changed lipid metabolism-related gene expression and affected the apoptosis pathway. To the best of our knowledge, this is the first study in which the effect of allantoin on the NASH disease and related mechanisms has been evaluated in an animal model.

Allantoin is known as an active compound in *yam, Dioscorea rhizome, *and some herbs [9] and has also been demonstrated as an imidazoline receptor Ι agonist (10). In this work, the MCD diet was used to induce NASH in the animals. Our histopathological findings showed that steatosis and hepatocyte ballooning after NASH induction and allantoin administration strikingly decreased lipid accumulation. Allantoin also lowered liver index, serum cholesterol, and LDL levels. Researchers have shown improvised effects of allantoin on hypertriglyceridemia and hypercholesterolemia in the cell line and animals (16). One of the main factors in NAFLD and NASH pathology is known to be endoplasmic reticulum stress (ER stress), which can promote steatosis in the hepatocytes (22). Findings of this study showed that allantoin attenuated GRP78 and ATF6, both of which play pivotal roles in the activation of ER stress. It has been previously demonstrated that naltrexone down-regulated GRP78 and ATF6 gene expression, alleviated ER stress, and improved liver steatosis in mice (19, 23). Other studies also showed improved effects of ER stress decrement in the NASH disease (24, 25). Herein, allantoin seems to ameliorate ER stress and lipid accumulation in the hepatic cells. 

Another feature of NASH pathology is insulin resistance and increased plasma glucose levels (26). Previous studies have reported that allantoin decreased plasma glucose and improved insulin sensitivity in type 1 diabetic rats (27). In another study, it has been shown that allantoin administration for three days increased GLUT4 mRNA and protein levels in muscles in STZ-diabetic rats (28, 29). Similarly, it was demonstrated that metformin decremented plasma glucose through activation of imidazoline receptor Ι in diabetic animals (30). Our results are consistent with previous reports, and it seems that there exists another mechanism for positive effects of allantoin on the reduction of steatosis, cholesterol, and LDL levels, probably owing to imidazoline receptor activation. Nevertheless, in our study, serum TG level did not decrease after allantoin treatment. This may be due to triglyceride transfer from the liver to plasma, which helps steatosis decrement in the liver.

Subsequently, to gain insight into the allantoin functions in lipid metabolism-related genes expression, SREBP1c, PPARα, Apo B, ACAT1, and FAS genes expression were assessed. SREBP1c and PPARα are two transcriptional factors related to lipid metabolism. Our results showed that allantoin down-regulated SREBP1c gene expression as a lipogenic factor rather than significantly increased PPARα gene expression as a lipolysis factor. FAS gene expression decreased while ApoB and ACAT1 (Thiolase I) increased after allantoin treatment. Apo B, ACAT1, and FAS are necessary enzymes for lipid metabolism and transfer. Numerous researchers have reported that decreased FAS levels are associated with liver steatosis improvement in MCD diet animals (31, 32). Upregulation of ApoB and ACAT1 could also lead to hepatic lipid accumulation decrement (20). Therefore, it seems that allantoin could be involved in SREBP1c and PPARα expressions and thereby change their downstream enzyme expression, promote TG and cholesterol metabolism, and lead to NASH improvement. Further studies could confirm this hypothesis. 

Inflammation has an essential role in NASH pathology. Lipid accumulation triggers inflammation pathways and plays a considerable role in NASH promotion (26). Our findings showed that allantoin administration ameliorated lobar inflammation, macrophage accumulation, ALT level, and TNFα both in gene expression and tissue levels. It has been shown that increased TNFα levels are associated with worsening NASH severity (33). Positive effects of resveratrol in the decrement of ALT and AST levels, TNFα gene expression, and NASH improvement has been reported (34). We previously also showed that naltrexone could attenuate ALT, AST, and TNFα levels in ER stress lipid steatosis (23). It has been revealed that allantoin decreased IgE, T helper 2 (Th2), IL4. and IL5 and has anti-inflammatory properties (13,14). Overall, it is highly probable that these findings indicate that treatment with allantoin diminishes liver inflammation and leads to NSAH improvement. However, in our experiments, IL6 gene expression did not decrease after allantoin administration. Previous studies have suggested the controversial roles of IL6 in the NASH pathology. One study reported the protective effect of IL6 blockade in the MCD diet-fed mice (35). The same group has shown that treatment with anti-mouse receptor antibody (MR16-1) increased plasma FFA and exacerbated hepatocyte apoptosis and fibrosis (36). In this context, it has been stated that increased cytokines levels could also lead to insulin resistance and hepatic steatosis and worsen NASH (26). These contradictions may be due to different interactions between lipids metabolism-related genes and IL6 and other cytokines. Nevertheless, more evidence is required to support this hypothesis.

To the best of our knowledge, apoptosis is an untreated problem in the NASH disease and NASH is associated with different apoptosis scores. Increased P53, Bax/Bcl2 ratio, and caspase3 indicate apoptosis progression. Many studies have reported that fatty acids accumulation is hepatotoxic and vast β-oxidation processes hyperactivate mitochondrial electron transfer chain, injure DNA, and promote apoptosis (26, 37). The current study showed that allantoin significantly decreased P53, Bax/Bcl2 ratio, and caspase3 mRNA expression. In the only study, it was shown that allantoin increased Bcl2 and decreased caspase3 expressions in STZ-treated β-cells and rats (38). Therefore, allantoin treatment probably attenuates apoptosis through down-regulation of P53 and decrease of Bax/Bcl2 levels. The decrease of mitochondrial membrane permeability and cytochrome c release lead to the decrement of caspase3 mRNA expression. However, the precise mechanism remains to be elucidated.

NASH: Non-alcoholic steatohepatitis

NASH: Non-alcoholic steatohepatitis

**Table 1:** Histopathological findings in experimental groups

	**control**	**allantoin**	**NASH**	**NASH+allantoin**
**Steatosis (%)**				
Grade 0	100(6)	83.3(5)		66(4)^#^
Grade 1		16.6(1)		33(2)
Grade 2				
Grade 3			100(6)*	
**Ballooning (%)**				
Grade 0	100(6)	100(6)		83.3(5)^#^
Grade 1			100(6)*	16.6(1)
**Lobar inflammation (%)**				
Grade 0	100(6)	100(6)		66(4)^ #^
Grade 1				33(2)
Grade 2			100(6)*	

*compared with the control group, #compared with the NASH group (Chi-square test)

NASH: Non-alcoholic steatohepatitis

**Table 2:** Liver index and serum biochemical analysis in different experimental groups

	**Liver index (%)**	**ALT(U/L)**	**TG(mg/dl)**	**Cholesterol (mg/dl)**	**LDL(mg/dl)**
**Control**	7.67± 0.17	14.33±1.2	110.50±5.86	93.66±5.36	8.06±1.07
**Allantoin**	8.25± 0.66	6.66±1.45	119.33±9.87	90.66±0.88	10.63±0.37
**NASH**	11.62± 0.53^*^	47.66±3.71^*^	45.00±6.15^*^	113.33±6.69^*^	17.90±0.26^*^
**NASH+allantoin**	8.26± 0.23^#^	22.33±3.75^#^	104.25±5.48^#^	89.25±1.93^#^	15.26±1.45^#^

* *P*<0.01 compared with the control group, # *P*<0.01 compared with the NASH group, (one-way ANOVA followed by Tukey’s *post hoc* test)

NASH: Non-alcoholic steatohepatitis; ALT: alanine aminotransferase; LDL: low-density lipoprotein

NASH: Non-alcoholic steatohepatitis

NASH: Non-alcoholic steatohepatitis

NASH: Non-alcoholic steatohepatitis

## Conclusion

In summary, our study demonstrated that allantoin could improve steatosis, hepatic inflammation, and injuries and plays a considerable role in ER stress decrement, lipid metabolism-related transcriptional factors, enzymes expression, and reduction of apoptosis agents in animal induced NASH. However, more studies are needed to clarify additional mechanisms by which allantoin alleviate NASH in mice.
